# Evolution of Virulence in Emerging Epidemics

**DOI:** 10.1371/journal.ppat.1003209

**Published:** 2013-03-14

**Authors:** Thomas W. Berngruber, Rémy Froissart, Marc Choisy, Sylvain Gandon

**Affiliations:** 1 Centre d'Ecologie Fonctionnelle et Evolutive (CEFE) - UMR 5175, Montpellier, France; 2 Laboratoire Biologie & Génétique des Interactions Plantes-Parasites (BGPI), UMR 385 INRA-CIRAD-SupAgro, Campus international de Baillarguet, Montpellier, France; 3 Laboratoire Maladies Infectieuses et Vecteurs : Ecologie, Génétique, Evolution et Contrôle (MIVEGEC), UMR 5290 CNRS-IRD-Université de Montpellier I-Université de Montpellier II, Montpellier, France; 4 Oxford University Clinical Research Unit, Hanoi, Vietnam; University College London, United Kingdom

## Abstract

Theory predicts that selection for pathogen virulence and horizontal transmission is highest at the onset of an epidemic but decreases thereafter, as the epidemic depletes the pool of susceptible hosts. We tested this prediction by tracking the competition between the latent bacteriophage λ and its virulent mutant λcI857 throughout experimental epidemics taking place in continuous cultures of *Escherichia coli*. As expected, the virulent λcI857 is strongly favored in the early stage of the epidemic, but loses competition with the latent virus as prevalence increases. We show that the observed transient selection for virulence and horizontal transmission can be fully explained within the framework of evolutionary epidemiology theory. This experimental validation of our predictions is a key step towards a predictive theory for the evolution of virulence in emerging infectious diseases.

## Author Summary

Why are some pathogens more virulent than others? Theory predicts that pathogens that ‘keep their host alive’ can sometimes outcompete virulent pathogens in times when transmission to new susceptible hosts is unlikely. Yet, this prospect of finding a new susceptible host changes itself throughout an epidemic. In the early stage of an epidemic susceptible hosts are abundant and virulent pathogens that invest more into horizontal transmission should win the competition. Later on, the spread of the infection reduces the pool of susceptible hosts and may reverse the selection on virulence. This may favor benign pathogens after the acute phase of the epidemic. We model this transient benefit for virulence and predict both the epidemiology and the evolution of pathogens during an epidemic. To put these predictions to the test we monitor the competition of the temperate bacterial virus λ and its virulent mutant λcI857 in experimental epidemics. Our experimental results agree remarkably well with all our theoretical predictions. This demonstrates the ability of evolutionary epidemiology to predict selection for virulence in an ongoing epidemic.

## Introduction

Understanding and predicting the conditions under which pathogens evolve towards higher levels of virulence (pathogen induced host mortality) is a major challenge in the control of infectious diseases [1], [2]. Nevertheless, the theoretical understanding of virulence evolution is often based on several major simplifying assumptions. In particular, the classical adaptive dynamics framework assumes that mutations are rare and thus that evolution occurs on a much slower time scale than epidemiological dynamics [2]. In other words, adaptive dynamics theory relies on the assumption that there is very little amount of genetic variation in the pathogen population and that a single pathogen strain reaches an equilibrium before a new strain arises by mutation. However, ecological and evolutionary time scales may overlap when the amount of genetic variation is high [3]–[5]. This is the case for many pathogens, and in particular for viruses with large mutation rates. The recurrent introduction of new mutants violates a major assumption of adaptive dynamics since many different strains may compete with each other before the system reaches a new endemic equilibrium [6]–[10]. Previous theoretical analyses suggest that the outcome of this competition changes strikingly throughout an epidemic; even though selection can act against virulent mutants at the endemic equilibrium, there is a transitory phase during the early stage of the epidemic where the abundance of susceptible hosts can favor the more transmissible and aggressive strains [11]–[18].

**Figure 1:**
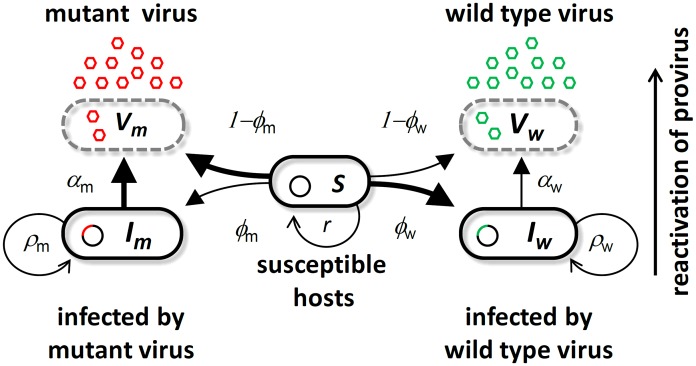
Schematic representation of the bacteriophage λ life cycle. Free viral particles of the wild type virus 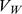 (green) and the virulent mutant 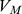 (red) infect susceptible cells 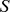. A proportion of successful infections leads to genome integration at rate 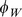 and 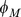 to produce infected cells 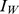 and 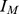 or results in cell lysis at rate 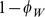 and 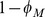. Infected cells lyse through spontaneous reactivation of the provirus at rate 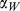 and 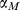 for 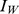 and 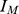, respectively. (See Table S1 in Text S1 for the definition and the values of all the parameters of this model).

Studying selection on virulence in the field is notoriously difficult because the characterization of the pathogen phenotypes can be obscured by host heterogeneity and healthcare measures. The unambiguous demonstration of the evolution of virulence evolution during an epidemic requires an experimental approach. Two different types of experimental setups can be used [19]. First, in a top-down approach, the evolution of the pathogen population is monitored in different environments (e.g. before and after an epidemic). In this case, making quantitative predictions on the epidemiology and evolution of the pathogen remains out of reach because evolutionary trajectories rely on random mutations occurring during the experiment. In contrast, the bottom-up approach attempts to measure and/or manipulate the initial amount of genetic variation in the pathogen population and try to predict the evolution from this standing genetic variation. Although many stochastic factors like new mutations may alter the quality of the predictions in the long term, this approach may provide good quantitative predictions in the short term. For this reason, we follow the bottom-up approach to analyze the interplay between the epidemiology and the evolution of the bacteriophage λ. To study the dynamics of selection on virulence we monitor the competition of the bacteriophage λ and its virulent mutant λcI857 throughout the development of an epidemic in continuous cultures of *E. coli*. Bacteriophage λ is a typical temperate virus which integrates into the host genome and transmits vertically to daughter cells at cell division. Integration of phage λ into the genome protects the host cell against superinfection of other λ phage particles and this way provides lifelong immunity to superinfection by other λ phage particles [20]. Nevertheless stochastic reactivation of the integrated phage results in lysis and destruction of the host cell, causing pathogen induced host mortality. Lysis of its host prevents vertical transmission but allows the phage to be transmitted horizontally to uninfected susceptible cells. Whereas the non-virulent λ wildtype transmits mostly vertically by dormant integration into the host genome, the virulent mutant λcI857 transmits mostly horizontally by host lysis (see Figure 1). This difference in virulence and transmission mode is the result of a point mutation in the λ virulence repressor protein cI which actively controls the decision to ‘kill or not to kill’ the host cell [21]–[23]. This active control of the fate of the infected cell has been shown to respond rapidly to different selection regimes [24]. Studying the competition between such cI variants is particularly relevant to study phage evolution during and epidemic.

In order to predict the competition between the temperate λ and the virulent λcI857 we first measured the effect of the cI857 mutation on several aspects of the viral life-cycle. In particular we focused on the effects of the cI857 mutation on the life-history traits known to be under the direct control of protein cI. We thus measured 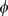 the ability to integrate into the genome of its host after infection, and 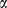 the spontaneous lysis rate of the lysogenic bacteria for both the λ wildtype and the mutant λcI857 (Figure S3). We used these life-history estimates and other estimates from the literature to parameterize an evolutionary epidemiology model which generated three clear-cut predictions. Our evolutionary experiments confirmed all three predictions and thus demonstrate the predictive power of evolutionary epidemiology theory.

## Results

### Evolutionary epidemiology theory

We modeled the competition of the temperate bacteriophage λ with its virulent mutant λcI857 throughout the course of an epidemic in chemostat cultures of its bacterial host *E. coli*. To understand and predict the competition dynamics of these two viruses throughout an epidemic we first developed a mathematical model. The epidemiology of phage λ can be described by the following set of ordinary differential equations for the densities of susceptible hosts, 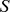, infected hosts, 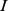, and free viral particles, 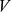:
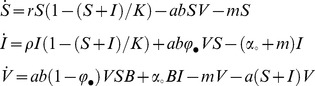
(1)where susceptible hosts and infected hosts grow at rate 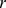 and 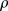, respectively, to a carrying capacity 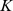 and die at a background mortality rate 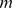. Infected hosts spontaneously switch to lysis at rate 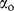 and produce 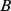 free viral particles. Viral particles adsorb to bacterial cells at rate 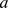, and inject their genome with probability 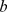. Injected viral genomes either replicate and destroy the host cell with probability 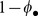 to release 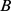 viral particles, or integrate into the host genome with probability 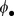. Integration of the virus into the genome of the host cell excludes superinfection by a second phage particle of the same kind [20]. Open and closed subscripts indicate averaged trait values taken over the provirus and the free virus stage, respectively (see section S1.2 in Text S1). To capture the evolution of viral traits we track the frequency of a strain 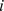 among all genome integrated provirus 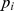, and among free viral particles 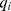 as follows (see section S1.2 in Text S1):
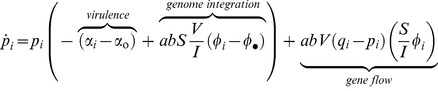
(2.1)

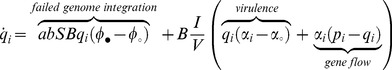
(2.2)


The above two equations readily show the different forces that affect the change in frequency of the virulent type in these two stages of the virus life cycle. First, in the provirus stage, the frequency of a virulent mutant decreases because of its increased lysis rate (first term in 2.1) and its lower rate of genome integration (second term in 2.1). But this frequency may increase by gene flow from the free virus stage (the last term in 2.1), because the frequency of the virulent mutant tends to be higher in the free virus stage (see prediction ii below). Second, in the free virus stage, the frequency of a virulent mutant increases because it has a lower rate of genome integration and a higher rate of lysis (first two terms in 2.2). Yet this frequency may decrease by gene flow from the provirus stage (last term in 2.2), because the frequency of the virulent mutant tends to be lower in the provirus stage (see prediction ii below).

**Figure 2:**
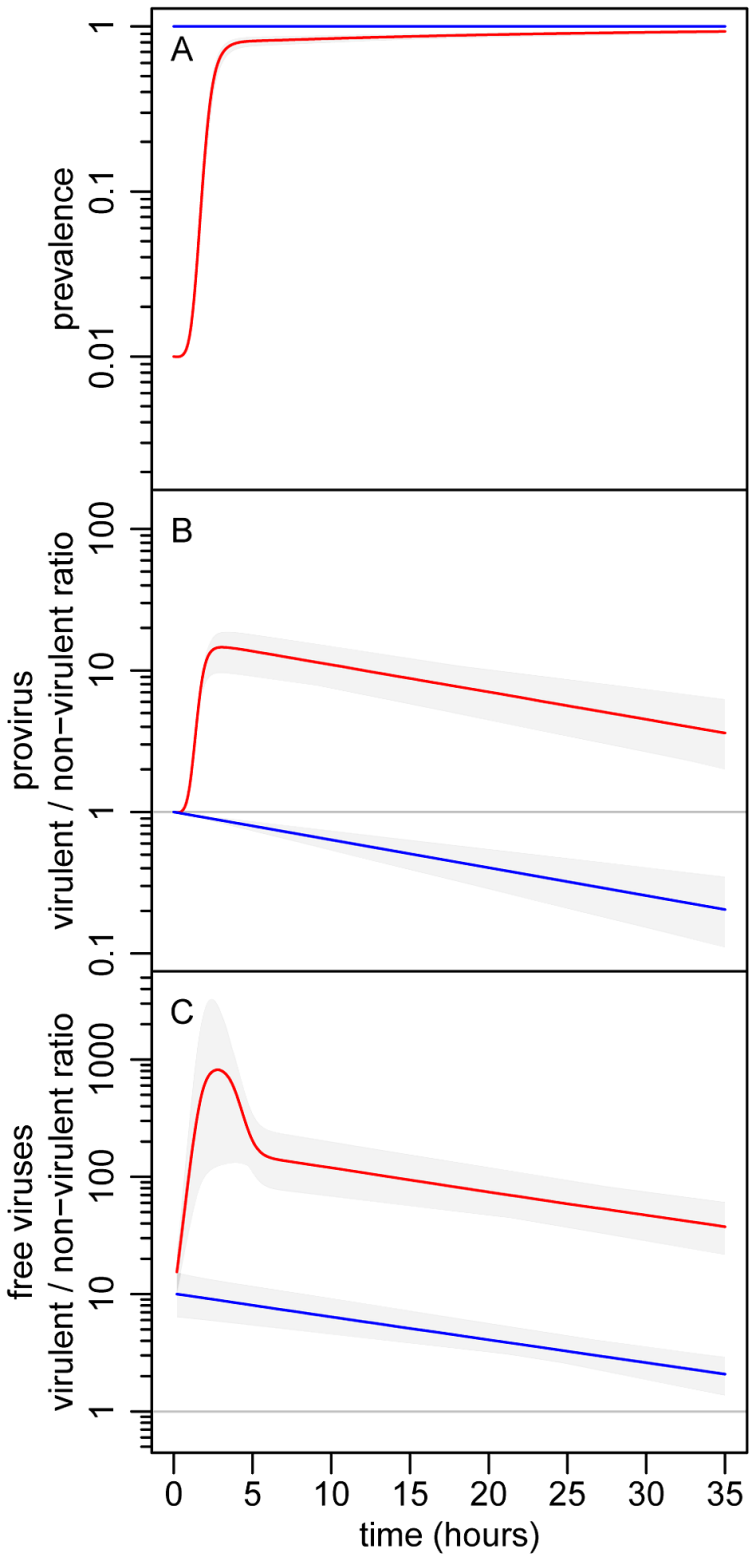
Theoretical evolutionary epidemiology. (A) change in prevalence (proportion of infected bacteria). (B) change in the λcI857/λ ratio in the provirus stage. (C) change in the λcI857/λ ratio in the free virus stage. The initial value of the λcI857/λ ratio in the provirus was 1∶1, and two initial prevalence values were considered: 1% (red) and 100% (blue). We ran 10000 simulations of our model (see equations (1) and (2)) allowing some variation over the phenotypic values (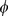 and 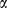) of the two virus strains. The gray envelopes show the range of variation among all simulation runs and colored lines show the median of these simulations (see section S1.3 in Text S1). See Table S1 in **supporting Text S1** for other parameter values.

Epidemiology, evolution and their interactions are fully integrated in the above five equations. Three general predictions emerge from the analysis of this model (see Figure 2): (i) The virulent mutant initially wins the competition with the wildtype when susceptible hosts are abundant, but the competitive outcome is reversed as soon as the epidemic reaches high prevalence; (ii) The virulent mutant is, at all times, more frequent among free viruses than among proviruses; and (iii) Lower initial prevalences result in a higher increase in virulence during the epidemic.

### Experimental evolutionary epidemiology

To test these three predictions, we infected *E. coli* chemostat cultures with a range of initial infection prevalences (between 1% and 100%) and monitored viral competition between λcI857 and λ (in a 1∶1 starting ratio at the provirus stage) during the spread of the epidemic. We introduced two fluorescent protein marker colors (CFP and YFP) into λcI857 and λ strains to measure their frequencies during competition. To experimentally control for a small marker color effect (see Table S2.1 in Text S1), we replicated the experiment switching marker colors between the two virus strains. We monitored strain frequencies in the provirus by flow cytometry, and in the free virus by marker specific qPCR (see section S2.1.2 in Text S1 and Figure S4).

In a first experiment, we tracked the change in prevalence (proportion of infected bacteria) and strain frequencies (in both the provirus and the free virus stages of the phage life cycle) by sampling hourly in 8 chemostats (2 marker/virulence combinations, 2 replicates and 2 initial prevalences: 1% and 100%). We performed a second experiment to further investigate the impact of initial prevalence using 6 chemostats (2 marker/virulence combinations and 3 initial prevalences: 1%, 10% and 99%; see section S2.3 in Text S1).

**Figure 3:**
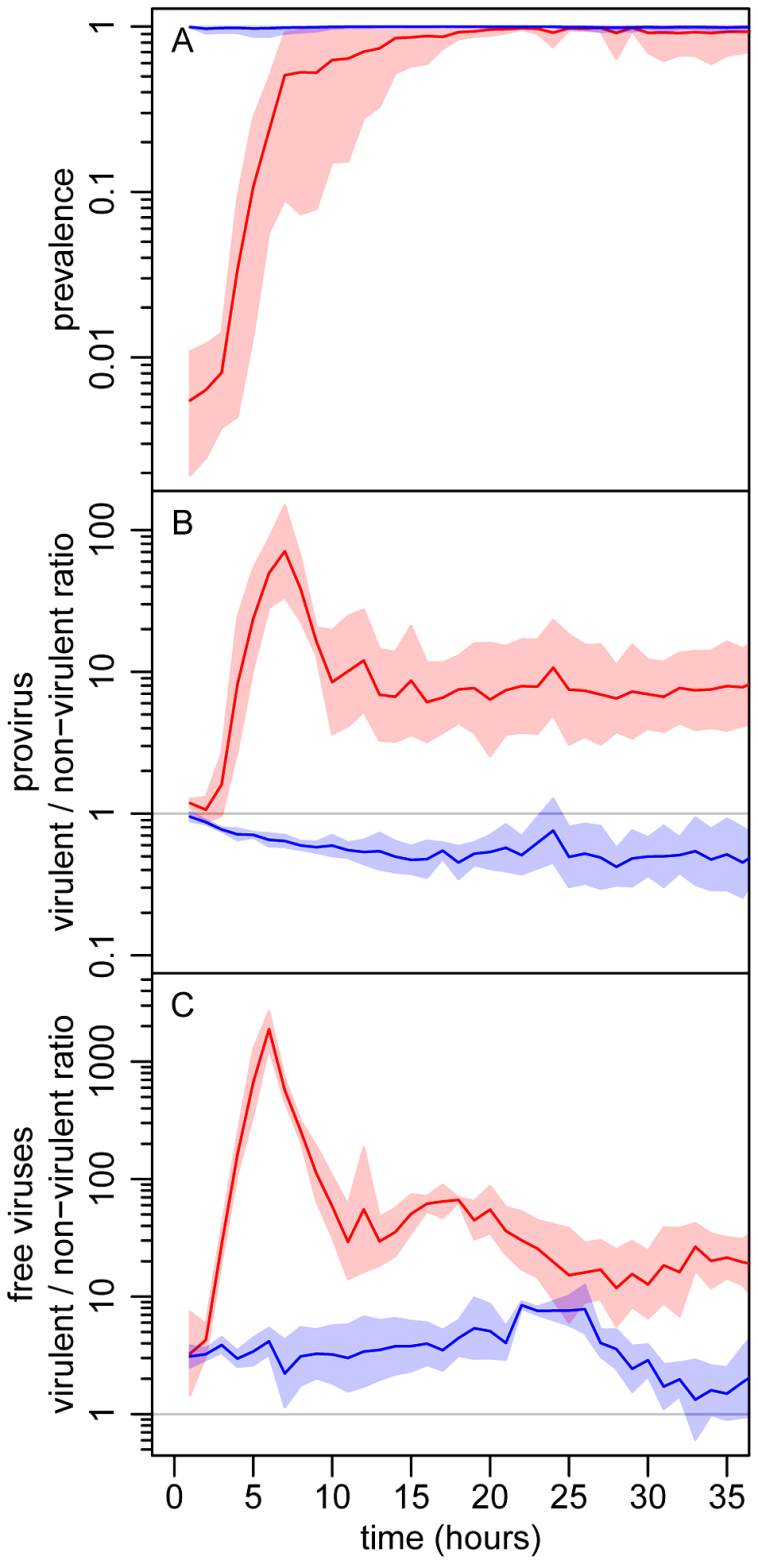
Experimental evolutionary epidemiology. (A) change in prevalence (proportion of infected bacteria). (B) change in the λcI857/λ ratio in the provirus stage. (C) change in the λcI857/λ ratio in the free virus stage. The initial value of the λcI857/λ ratio in the provirus was 1∶1, and competition was started from two initial prevalence values: 1% (red) and 100% (blue). The data was obtained from the first experiment. The lines are the mean over four chemostats (2 marker/virulence combinations with 2 independent replicates), and the envelopes show the 95% confidence intervals of the log transformed data.

**Figure 4:**
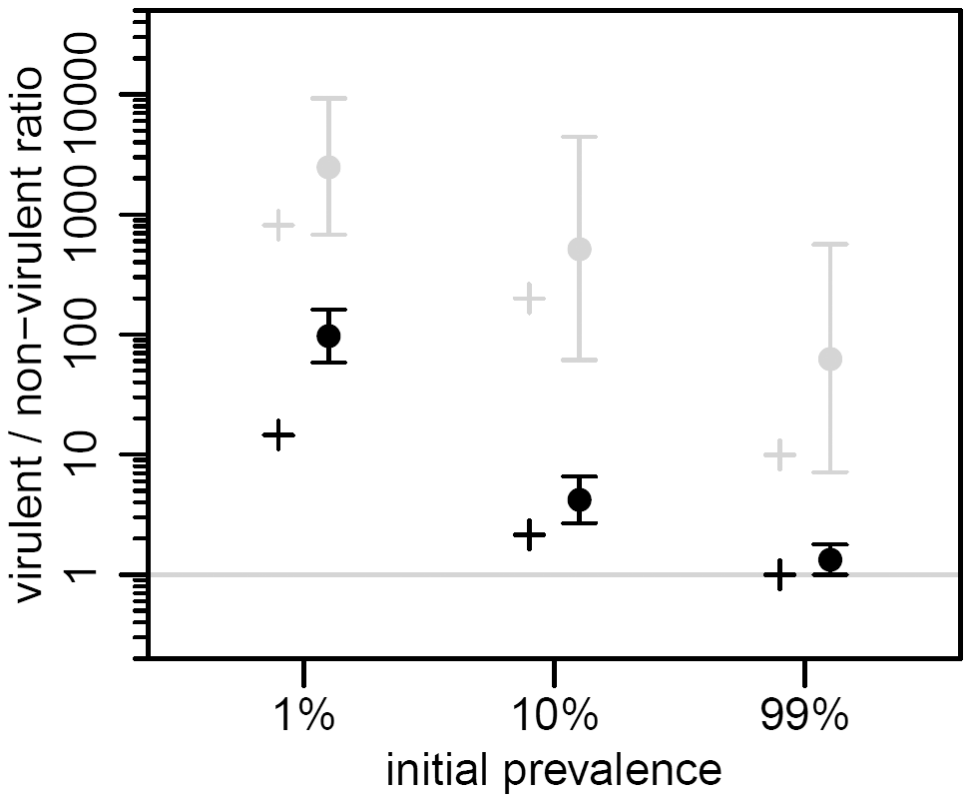
Effect of initial prevalence on transient virulence evolution, in the two life stages of the virus. We plot the maximal value of the λcI857/λ ratio from our theoretical model (crosses, same parameter values as in Figure 2) and from the second experiment (dots and vertical bars are the means and their 95% confidence intervals over two chemostats of the log transformed data, see supplementary information). The λcI857/λ ratio is shown for both the provirus (black) and the free virus (gray) stages. The λcI857/λ ratio is significantly higher among free viruses than among proviruses, and decreases significantly as the initial prevalence increases (maximal λcI857/λ ratio in the first 15 hours of the second experiment, see also Figure S6).

Starting from an initial prevalence of 1% the epidemic spread rapidly until nearly all hosts were infected, roughly 10 h later (Figure 3A). The virulent λcI857 rapidly outnumbered λ in both the provirus and the free virus compartments. Yet, despite this initial advantage in the first 7 h of the epidemic, the frequency of the virulent λcI857 started to decrease in both compartments thereafter (Figure 3 and Figure 4). This confirms our first theoretical prediction. Furthermore, the frequency of the virulent mutant λcI857 remained higher among free viruses than among proviruses during the entire course of the epidemics (Figure 3 and Figure 4). This confirms our second prediction. Moreover, as expected from our third prediction, at an initial prevalence of 100% the virulent mutant λcI857 lost the competition from the outset of the experiment (Figures 3B,C). The third prediction got additional support from the second experiment where the value of the peak frequency of the virulent mutant decreased with higher initial prevalence (Figure 4 and Figure S6).

## Discussion

To demonstrate that epidemiology can affect selection on viral virulence and transmission mode we studied the competition between two viral strains during experimental epidemics in chemostats. The two main life-history traits that govern virulence and transmission in λ (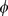 the ability to integrate into the genome of its host after infection, and 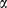 the lysis rate of the lysogenic bacteria) were measured for these two viral strains. We parameterized a model for the competition of several viral strains throughout an epidemic using estimations for the remaining parameter values from other published studies (Table S1 in Text S1). This model was used to generate three qualitative predictions on the epidemiology and the evolution of the bacteriophage λ. Our experimental results agree well with all three theoretical predictions. This demonstrates the predictive power of the bottom-up approach we used to model this system. This study confirms the importance of modeling both epidemiology and evolution to accurately predict the transient evolution of pathogens. In particular, the shift between positive and negative selection on the virulent mutant makes only sense because we took into account the feed-back of epidemiology on the evolution of the virus.

Our theoretical predictions are based on the competition between two pathogen variants in a fully susceptible host population. In this way we focus on the short-term evolutionary dynamics taking place during an epidemic. As pointed out above, the accuracy of these predictions is expected to drop as other mutations in virus or host come into play. In particular, compensatory mutations that reduce phage virulence could alter the ultimate trajectory of λcI857, as it would no longer pay the cost of virulence in the long run. In the present experiment, we did not find evidence of such compensation (Figure S5). Nevertheless, we can readily include compensation in our model to judge its effect on short and long-term dynamics. We found that if we allow for these compensatory mutations the above short term predictions still hold (Figure S1). Another evolutionary route the pathogen could take is to escape superinfection inhibition, which would allow it to gain access to hosts even when all the bacteria are infected [25]. However, in λ the rate of mutation towards such ultravirulent strains has been shown to be very small and is thus unlikely to affect the short-term evolutionary dynamics [26]. In the long term, however, the coevolution between superinfection inhibition and the resistance to superinfection inhibition may play a major role for the evolutionary maintenance of viral latency and the emergence of diversity in λ-like phages [27]. In addition, the *E. coli* host cell may also acquire resistance to λ by well known mechanisms [28]–[29]. We did find some evidence of host resistance evolution but only in the large volume chemostats (50 mL, second experiment) and not before 40 h (Figure S7 and Figure S8), which indicates that the appearance of these mutations is limited by population size and by time. Again, including host resistance in our model confirms that the above three qualitative predictions hold in the short-term (Figure S2).

Our model assumes that all the parameter values governing phage life cycle remain constant throughout the experiment. The lyzogenisation rate of phage λ, however, is known to vary with the multiplicity of infection (MOI), which is the number of virus entering the bacteria. The higher the MOI, the higher the lysogenization rate [22], [30]. For the sake of simplicity we do not consider this effect in our model but additional simulations indicate that it does not alter qualitatively our conclusions (not shown) because both the wild type and the cI857 mutant harbor this phenotypic plasticity [30]. Our model, however, may shed some light on the adaptive nature of the sensitivity of the rate of lysogenisation to the MOI. The MOI provides accurate information of the epidemiological state of the environment. When the MOI is low the number of susceptible hosts is likely to be high and it is a good strategy to lyse and to try infecting new hosts horizontally. In contrast, when the MOI becomes high, it is very unlikely that a free virus particle will encounter a susceptible host. In this case the phage would benefit more from investing into lyzogeny and vertical transmission [31]–[32]. In other words, the evolution of plasticity may be another evolutionary outcome resulting from the epidemiological feedback during an epidemic.

When do we expect epidemiology to feed back on the evolution of virulence? In our experimental system, this feedback operates because the spread of the virus in the population reduces the density of susceptible hosts. This erodes the benefit of virulence (horizontal transmission) while the cost of virulence (induced host mortality) remains. Note that this qualitative result is robust to variations of the initial frequency of the mutant strain (not shown). In our system, the cost of virulence acts mainly via the reduction of vertical transmission. Yet, in the absence of vertical transmission, a similar pattern is expected when horizontal transmission carries other costs. In many lytic phages these fitness costs result from the trade-off between lysis time and burst size [33]. The virus with short lysis time (and small burst size) may only outcompete the virus with longer lysis time (and larger burst size) at the beginning of the epidemic when the availability of susceptible bacteria is maximal [34]. Similar patterns of transient evolution are also expected in pathogens that transmit throughout the course of the infection. In this case, higher rates of horizontal transmission are often associated with more aggressive host exploitation strategies which reduce host life-span and pathogen's infectious period. Shortened infectious period can result in substantial fitness costs for the virulent pathogens. It is only during the early stages of an epidemic that such virulent strains may outcompete the others [3]–[6]. Hence, the transient evolution we report in our study is expected whenever there is a fitness cost associated with increased virulence and horizontal transmission.

Epidemic feedback on the evolution of virulence is likely to be widespread and could affect many other pathogens. For example, this feedback may also operate during viral invasion into a multicellular host organism (within-host evolution). During this within-host spread of the infection the availability of susceptible cells is expected to drop. Noteworthy this effect is particularly strong for viruses with superinfection exclusion like herpes- and retroviruses as well as phage λ, where infected cells remain resistant to a second infection and can vertically pass on this immunity to daughter cells [35]–[36]. A similar evolutionary trajectory is expected in large scale epidemics (between-host evolution) when the spread of the infection reduces the availability of susceptible hosts because both infected and recovered individuals tend to be immune to new infections [36], [37]. If multiple infections or superinfections are possible, the evolution of virulence results from selection acting on two different levels: within and between hosts [38]–[40]. Nevertheless it is possible to modify our model to allow for this additional level of complexity but the evolutionary outcome depends on the relative competitive abilities of different variants which can be obtained in some pathogens [40] and could thus be used to provide quantitative predictions when these two levels of selection are acting on the evolution of virulence.

Our experimental epidemics occur in a small and well-mixed community free of the considerable complications arising from stochasticity, multi-host life cycles, host immunity, input of new mutations and spatial structuring of host populations. Albeit a necessary simplification of a more complex reality, our experiments provide a unique opportunity to understand pathogen evolution during the course of an infection (within-host dynamics). In HIV and HCV, for instance, the replicative fitness and the ability of the virus to resist therapeutic drugs has been shown to change throughout the course of the infection [41]–[43]. The understanding of such within-host evolution is key to providing more effective therapies that are not necessarily aimed towards eradication of the pathogen but towards patient health improvement [44]–[45]. On a larger spatial scale, the interplay between epidemiology and evolution that we demonstrate here can have far reaching consequences for emergent epidemics during the spread into a host population (between-host dynamics) [6]. More specifically, we expect drastic changes of virulence between early and later stages of global pandemics, but also between different stages of the pathogen life cycle. Our joint theoretical and experimental approach is a first step towards a framework aiming to forecast both the epidemiological and evolutionary trajectories of pathogens.

## Materials and Methods

### Fluorescently labeled phage construction

The GFP-Kan cassette from λGFP (from reference [21]) was amplified with primers λ20165F (CGCAGAAGCGGCATCAGCAA) and λ22543R (GGACAGCAGGCCACTCAATA) and subcloned into pUC18. Subsequently GFP was mutated to CFP and YFP by a quick-change PCR approach with megaprimers amplified from pDH3 and pDH5 (Yeast Resource center, University of Washington, primers: Fwd TGGCCAACACTTGTCACTAC, Rev AGAAGGACCATGTGGTCTCT). CFP-Kan and YFP-Kan cassettes were integrated into the prophage of K12[λ] and KL740[λcI857] (Yale E.coli Stock Center) by the aid of recombineering plasmid pKM201 (Addgene). Fluorescent lysogens were induced by 10 µM MitomycinC and chloroformed lysate was used to reinfect *E. coli* MG1655.

### Life-history of fluorescently marked viral strains (Figure S3)

Our method for the detection of selection on virulence is based on the competition of the non-virulent λ wildtype against the virulent λcI857. In order to verify the expected differences in life-history traits between those strains and to obtain rough parameter estimates for the simulations we measured the viral life-history traits *virus production* (PFU/mL), *genome integration rate* (% lysogenized) and *vertical transmission* (CFU/mL). We determined these traits for all constructed viruses (λcI857CFP, λcI857YFP and λCFP, λYFP) by independent life-history assays prior to competition in the chemostat. The life-history traits were measured by the following three independent assays. (1) Virus production (PFU/mL) (Figure S3A) was determined by growing lysogen cultures to OD600 nm = 0.6 at 30°C and shifting them to 35°C and 38°C for 2 h until lysis occurred. From these lysates, viral titers were determined by qPCR on a Roche LightCycler480 (primers F:5′AATGAAGGCAGGAAGTA3′ R:5′GCTTTCCATTCCATCGG3′). Viral titers were calculated from a calibration curve based on CP values of a dilution series of a lysate of λvir of known titer (3×109 pfu). (2) Vertical transmission (CFU/mL) (Figure S3A) was measured by diluting lysogen cultures of λCFP, λYFP and λcI857CFP, λcI857YFP to OD600 nm = 0.07 and growing them for 6 h at 35°C and 38°C in eight replicates each in 96-well plates on a Titramax shaker (Heidolph, Germany) at 900 rpm. Every hour OD600 nm was measured in an Infinity200 microplate reader (Tecan, Austria). OD600 nm values were converted to CFU's by a calibration curve which was obtained by plating. (3) Lysogenization rate (Figure S3B) was determined by challenging non-infected E.coli MG1655 with 10^8^ PFU/mL free virus particles of λCFP, λYFP, λcI857CFP and λcI857YFP for 24 h. After 24 h, the proportion of lysogenized (fluorescent) cells was determined by flow cytometry.

### Chemostat growth conditions

Media, 0.25 x LB, 0.2% Maltose, 10 mM MgSO4 and 5 mM IPTG. Dilution rate 0.8/h with 5 mL chamber volume at 35°C (50 mL chamber volume in the second experiment). Samples were drawn at 1 h intervals and stored at 2°C in 10 mM Na-citrate.

### Quantifying competition in the provirus stage by flow cytometry (Figure 2B and 3)

To follow the relative prevalence of each strain in experimental epidemics we distinguished cells infected by CFP and YFP tagged virus through flow cytometry. CFP was detected at 405 nm excitation and 510/50BP emission and YFP was detected at 488 nm excitation and 530/30BP emission on a BD FacsCantoII flowcytometer. We used the FlowJo7 (Tree Star, Inc.) software to apply automatic compensation and gating.

### Quantifying competition in the free virus stage by marker specific qPCR (Figure 2C and 3)

To quantify the amount of each strain in the free virus stage we developed CFP and YFP specific qPCR primer sets that match the functional substitution T203Y, which is responsible for the spectral shift from GFP to YFP and V163A and N164H which are used as fluorescence enhancers of CFP (Table S3 in Text S1). Our primers show no non-specific amplification even in large excess of the non-specific template (Figure S4) but high amplification efficiencies (AE) on their specific template (AE = 2.0 for CFP and AE = 1.98 for YFP).

## Supporting Information

Figure S1
**Theoretical evolutionary epidemiology (analogous to** **Figure 2** **) for a modified model which allows for virulence compensation.** (A) change in prevalence (proportion of infected bacteria). (B) change in the λcI857/λ ratio in the provirus stage. (C) change in the λcI857/λ ratio in the free virus stage. The virus mutation probability on virulence is 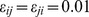. See Table S1 in Text S1 for other parameter values. (Red and blue line: 1% and 10% initial prevalence. Gray envelopes show the range of variation among the 10000 simulation runs and colored lines show their median).(TIF)

Figure S2
**Theoretical evolutionary epidemiology (analogous to** **Figure 2** **) for a modified model which allows for mutation towards host resistance.** (A) change in prevalence (proportion of infected bacteria). (B) change in the λcI857/λ ratio in the provirus stage. (C) change in the λcI857/λ ratio in the free virus stage. The host mutation probability towards resistance is 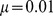, and the cost of resistance is assumed to be 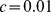. See Table S1 in Text S1 for other parameter values. (Legend similar to Figure S1, except black line: frequency of resistant cells).(TIF)

Figure S3 **Life-history of the constructed viral strains λcI857CFP, λcI857YFP and λCFP, λYFP.** (**A**) **Horizontal (free phages PFU/mL) and vertical transmission (infected cells CFU/mL).** At 35°C: λcI857CFP and λcI857YFP show significantly higher horizontal transmission and reduced vertical transmission compared to the wildtype constructs λCFP and λYFP. At 38°C, horizontal transmission of λcI857CFP and λcI857YFP is further increased and vertical transmission is further reduced. (**B**) **Genome integration rate (% lysogenized cells at 24 h).** Lysogenization at 35°C is about 6 fold higher for λCFP, λYFP than for the mutants λcI857CFP and λcI857YFP ((A) Crosses, 95% CI. (B) Bars, 95% CI. Gray circles, raw data, see section S2.1.1 in Text S1 for statistical analysis).
(TIF)

Figure S4 **Test for cross-specificity of CFP and YFP specific qPCR primers.** Primers at 1 µM concentration were tested on dilution series of pure λCFP and λYFP lysates (10^9^ to 10^2^ pfu in 10-fold steps) as well as a reciprocal mixture of the dilutions series (5×10^8^ : 5×10^1^ to 5×10^1^ : 5×10^8^ pfu/mL of λCFP : λYFP). qPCR on the reciprocal lysate mixtures shows no non-specific quantification even with a 10^7^ fold excess of the non-specific template (see Table S3 in Text S1 for primers).
(TIF)

Figure S5 **Test for the occurrence of mutations that compensate virulence.** As a proxy for virulence we calculated viruses produced per infected cell (viruses/cell) for the λcI857 mutant strains and the wildtype strains. The λcI857 produces more viruses/cell and is more virulent than the wildtype as long as the ratio (viruses/cell)_mutant_ devided by (viruses/cell)_wildtype_ is larger than 1. Indeed this ratio is significantly larger than 1 throughout the experiment (except t = 25 and 33 h in the 1% treatment). Hence, the λcI857 mutants have remained more virulent than the wildtype even if compensatory mutations might have occurred. (blue area: 1% initial prevalence, red area: 100% initial prevalence, solid line and shading: Mean and 95% CI envelope of the log transformed data from 4 chemostats).
(TIF)

Figure S6 **Competition dynamics in the second chemostat experiment with initial prevalence of 1%, 10% and 99%.** (A) Prevalence, (B) fitness benefit for the provirus and (C) free virus in the second chemostat experiment. The 1% initial prevalence treatment (blue) leads to the highest benefit of virulence. This benefit of virulence decreases in the 10% and 99% initial prevalence (green and red). The maxima of B and C between t = 0 and t = 15 h were extracted to create Figure 4 in the main text (Solid lines: Mean, shading: 95% CI interval of log transformed data from 2 chemostats pooled by color replicate).
(TIFF)

Figure S7 **Invasion of resistant host cells.** In the second experiment, resistant, but non-infected, host cells invaded 5 out of 6 chemostats after 40 h and caused a drop in overall prevalence. Chemostat 5 (red solid line) showed no invasion of resistant cells and maintained high prevalence. (Solid lines: λCFP versus λcI857YFP and dotted lines λYFP versus λcI857CFP. Blue, green and red: 1%, 10% and 99% initial prevalence, black numbers correspond to the numbering in Figure S8).
(TIF)

Figure S8 **Test for the origin of resistance.** We cross-streaked individual colonies from t = 60 h of each chemostat against the indicator strain λKH54h80ΔcI (this strain enters through the FhuA receptor and only lyses non-lysogens). Resistant hosts from chemostats 1, 2, 3, 4 and 6 were sensitive to λKH54h80ΔcI and are therefore non-lysogens and most likely lamB resistance mutants. Since most colonies from chemostat 5 carry a prophage they are not lysed by the indicator strain.
(TIF)

Text S1 **Supporting text file containing the theoretical appendix, supporting experiments and statistics.**
(PDF)
