# Supplementary figures and images for: Evolution of Virulence in Emerging Epidemics
Source: PLoS Pathog. 2013 Mar 14;9(3):e1003209. doi: 10.1371/journal.ppat.1003209 (PMC3597519; doi:10.1371/journal.ppat.1003209)

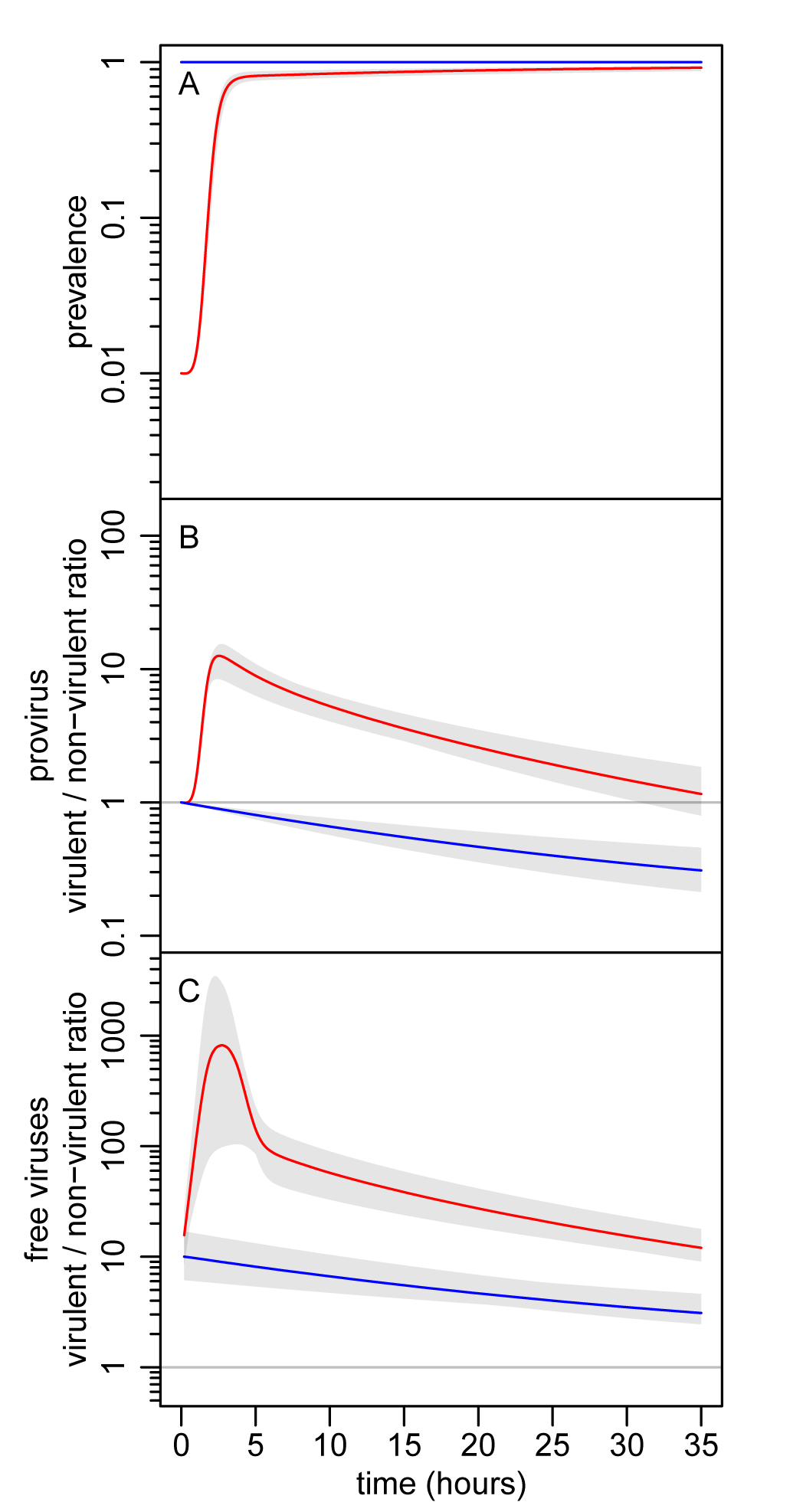

Supplement: Figure S1 — Theoretical evolutionary epidemiology (analogous to Figure 2 ) for a modified model which allows for virulence compensation. (A) change in prevalence (proportion of infected bacteria). (B) change in the λcI857/λ ratio in the provirus stage. (C) change in the λcI857/λ ratio in the free virus stage. The virus mutation probability on virulence is . See Table S1 in Text S1 for other parameter values. (Red and blue line: 1% and 10% initial prevalence. Gray envelopes show the range of variation among the 10000 simulation runs and colored lines show their median). (TIF) [file ppat.1003209.s001.tif]

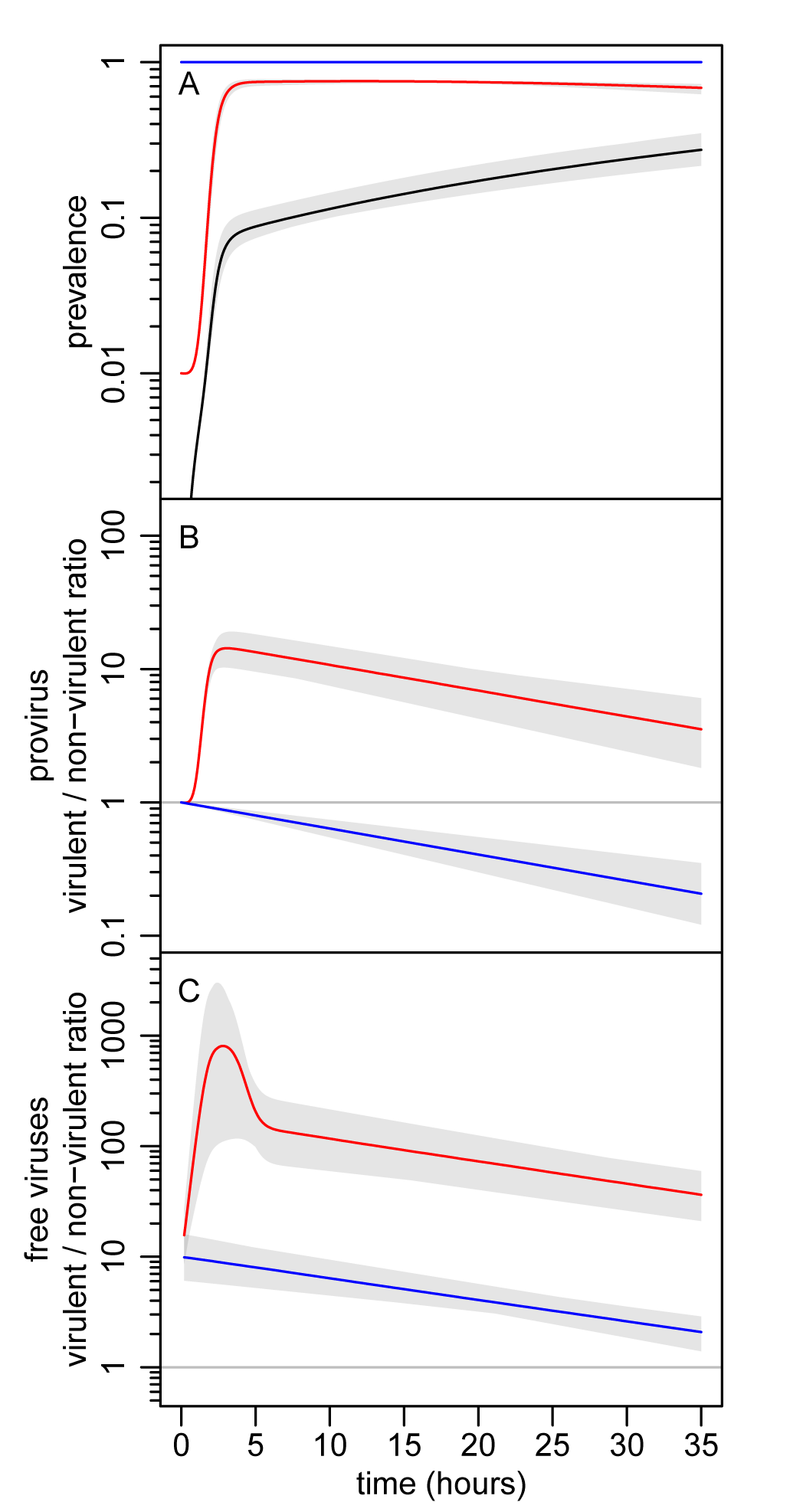

Supplement: Figure S2 — Theoretical evolutionary epidemiology (analogous to Figure 2 ) for a modified model which allows for mutation towards host resistance. (A) change in prevalence (proportion of infected bacteria). (B) change in the λcI857/λ ratio in the provirus stage. (C) change in the λcI857/λ ratio in the free virus stage. The host mutation probability towards resistance is , and the cost of resistance is assumed to be . See Table S1 in Text S1 for other parameter values. (Legend similar to Figure S1, except black line: frequency of resistant cells). (TIF) [file ppat.1003209.s002.tif]

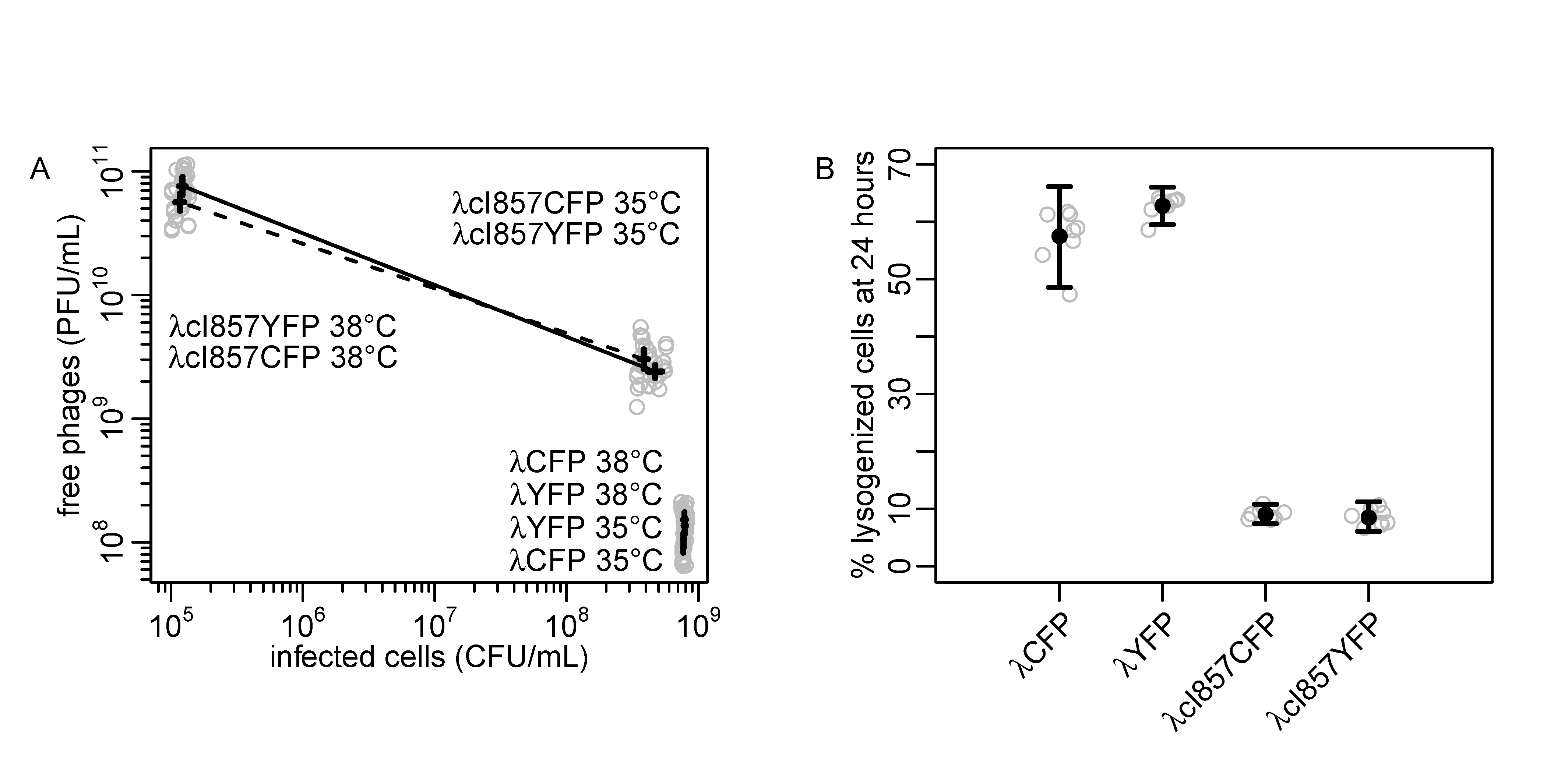

Supplement: Figure S3 — Life-history of the constructed viral strains λcI857CFP, λcI857YFP and λCFP, λYFP. (A) Horizontal (free phages PFU/mL) and vertical transmission (infected cells CFU/mL). At 35°C: λcI857CFP and λcI857YFP show significantly higher horizontal transmission and reduced vertical transmission compared to the wildtype constructs λCFP and λYFP. At 38°C, horizontal transmission of λcI857CFP and λcI857YFP is further increased and vertical transmission is further reduced. (B) Genome integration rate (% lysogenized cells at 24 h). Lysogenization at 35°C is about 6 fold higher for λCFP, λYFP than for the mutants λcI857CFP and λcI857YFP ((A) Crosses, 95% CI. (B) Bars, 95% CI. Gray circles, raw data, see section S2.1.1 in Text S1 for statistical analysis). (TIF) [file ppat.1003209.s003.tif]

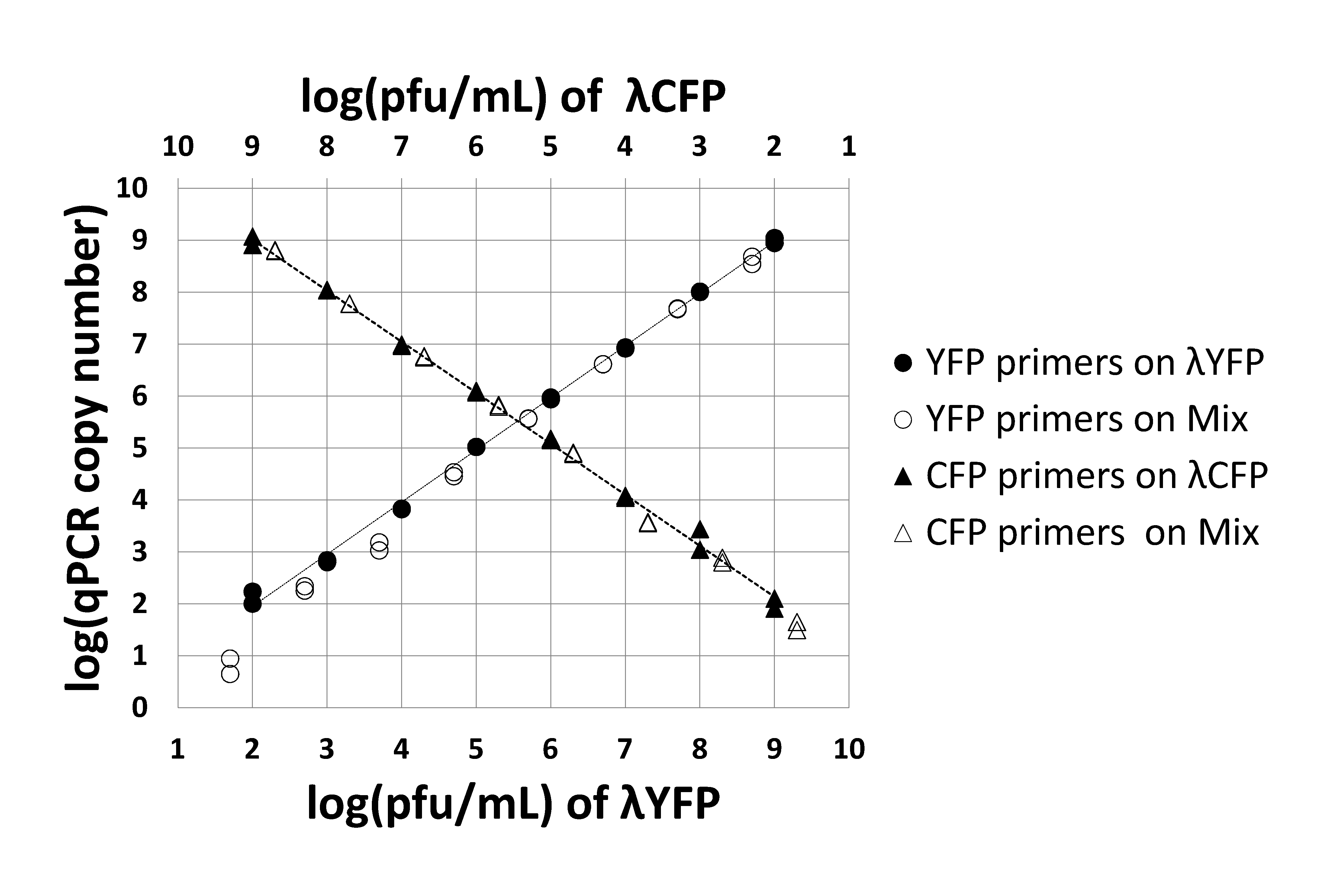

Supplement: Figure S4 — Test for cross-specificity of CFP and YFP specific qPCR primers. Primers at 1 µM concentration were tested on dilution series of pure λCFP and λYFP lysates (109 to 102 pfu in 10-fold steps) as well as a reciprocal mixture of the dilutions series (5×10^8^ : 5×101 to 5×101 : 5×10^8^ pfu/mL of λCFP : λYFP). qPCR on the reciprocal lysate mixtures shows no non-specific quantification even with a 107 fold excess of the non-specific template (see Table S3 in Text S1 for primers). (TIF) [file ppat.1003209.s004.tif]

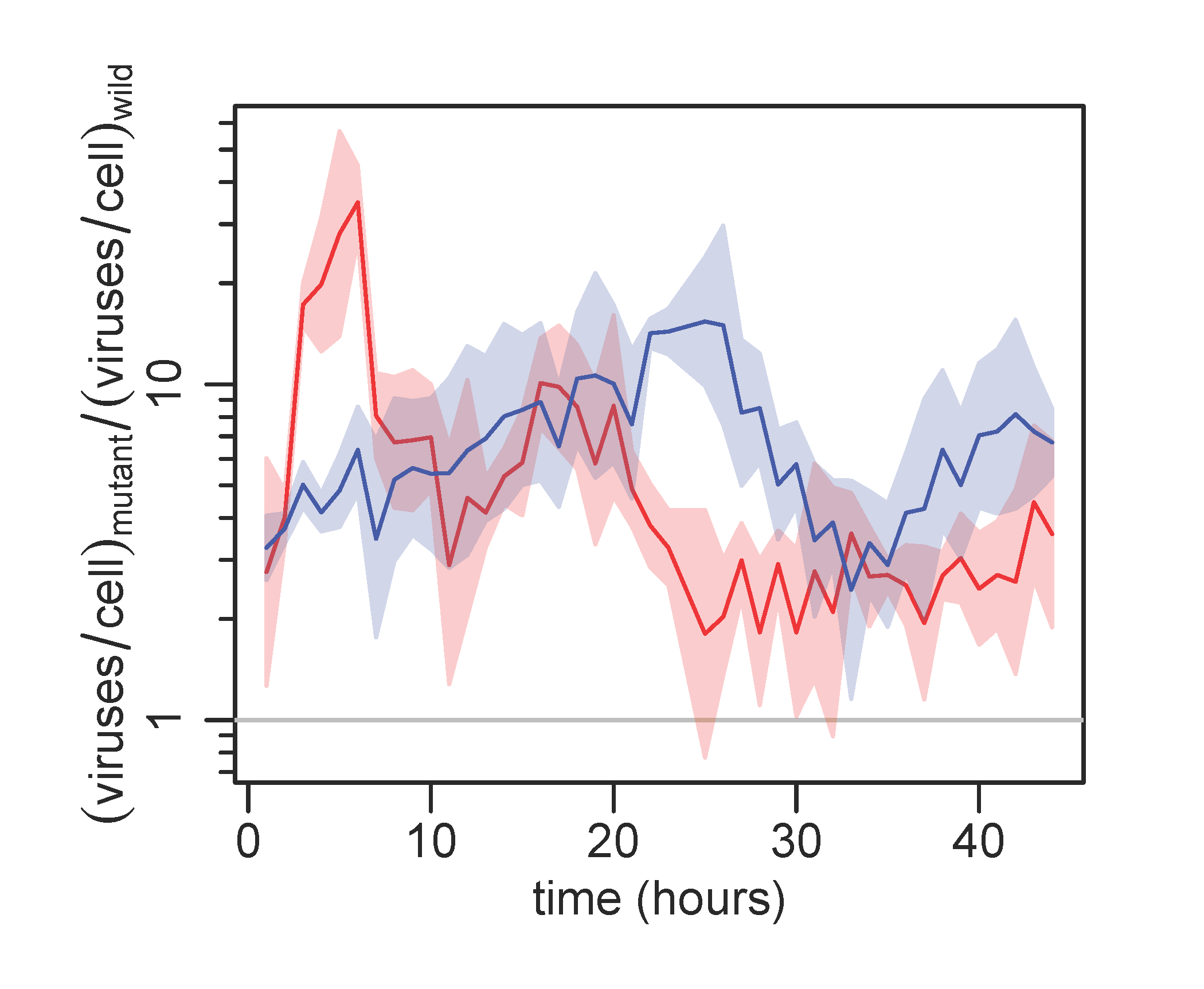

Supplement: Figure S5 — Test for the occurrence of mutations that compensate virulence. As a proxy for virulence we calculated viruses produced per infected cell (viruses/cell) for the λcI857 mutant strains and the wildtype strains. The λcI857 produces more viruses/cell and is more virulent than the wildtype as long as the ratio (viruses/cell)mutant devided by (viruses/cell)wildtype is larger than 1. Indeed this ratio is significantly larger than 1 throughout the experiment (except t = 25 and 33 h in the 1% treatment). Hence, the λcI857 mutants have remained more virulent than the wildtype even if compensatory mutations might have occurred. (blue area: 1% initial prevalence, red area: 100% initial prevalence, solid line and shading: Mean and 95% CI envelope of the log transformed data from 4 chemostats). (TIF) [file ppat.1003209.s005.tif]

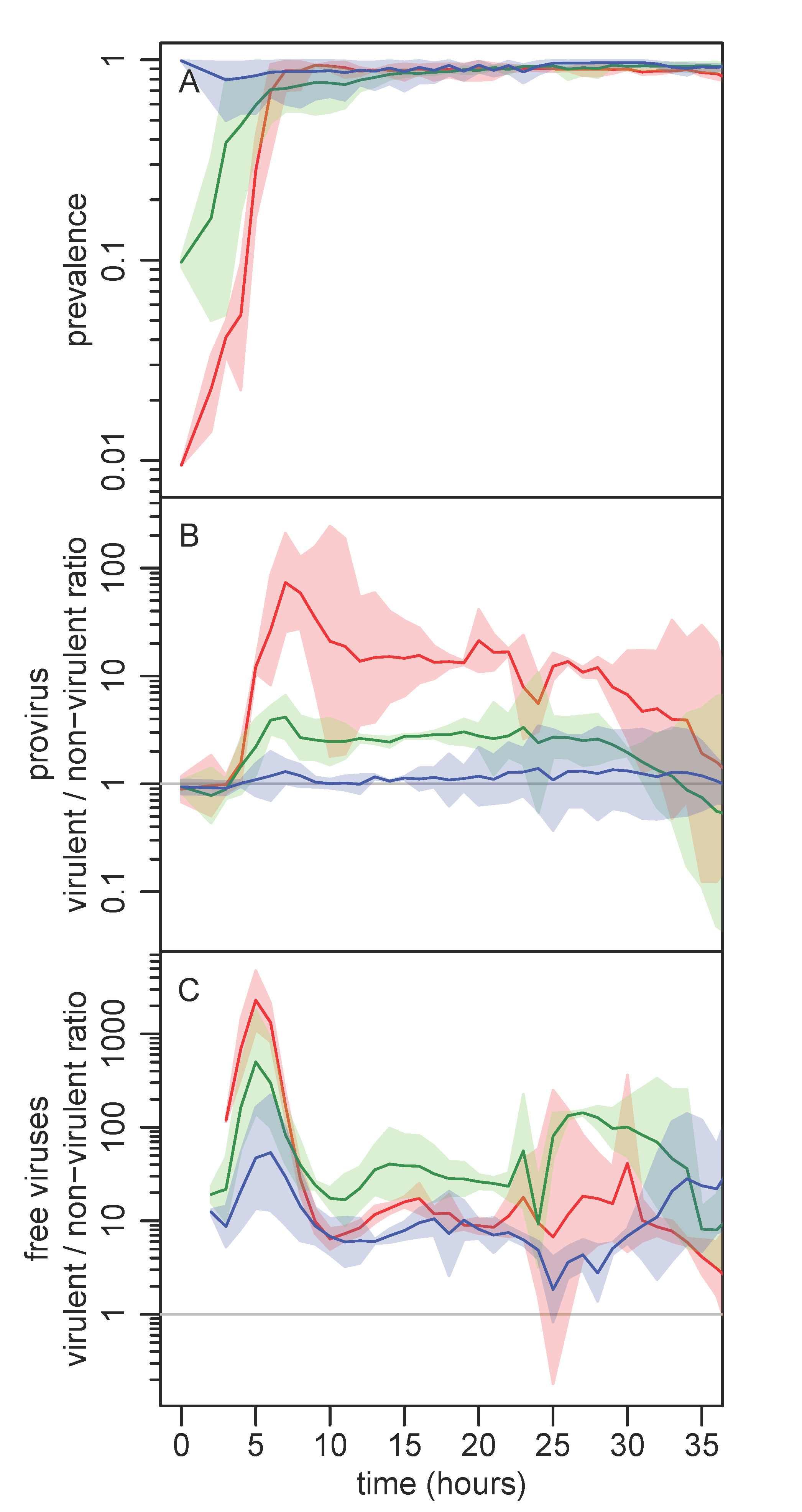

Supplement: Figure S6 — Competition dynamics in the second chemostat experiment with initial prevalence of 1%, 10% and 99%. (A) Prevalence, (B) fitness benefit for the provirus and (C) free virus in the second chemostat experiment. The 1% initial prevalence treatment (blue) leads to the highest benefit of virulence. This benefit of virulence decreases in the 10% and 99% initial prevalence (green and red). The maxima of B and C between t = 0 and t = 15 h were extracted to create Figure 4 in the main text (Solid lines: Mean, shading: 95% CI interval of log transformed data from 2 chemostats pooled by color replicate). (TIFF) [file ppat.1003209.s006.tiff]

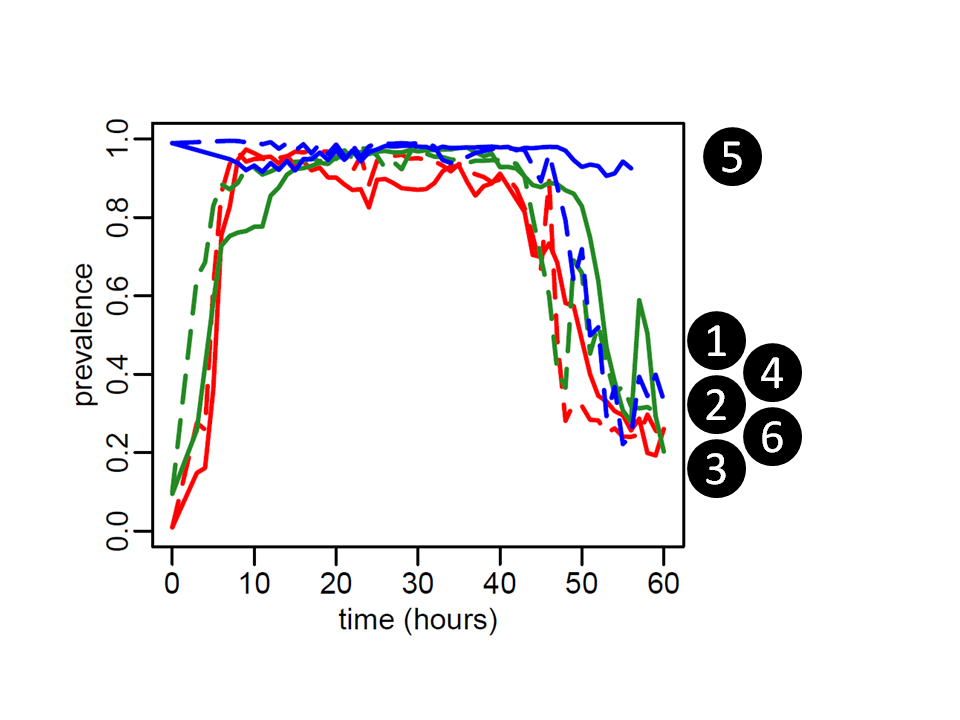

Supplement: Figure S7 — Invasion of resistant host cells. In the second experiment, resistant, but non-infected, host cells invaded 5 out of 6 chemostats after 40 h and caused a drop in overall prevalence. Chemostat 5 (red solid line) showed no invasion of resistant cells and maintained high prevalence. (Solid lines: λCFP versus λcI857YFP and dotted lines λYFP versus λcI857CFP. Blue, green and red: 1%, 10% and 99% initial prevalence, black numbers correspond to the numbering in Figure S8). (TIF) [file ppat.1003209.s007.tif]
