# Supplementary material for: Evolution of Virulence in Emerging Epidemics
Source: PLoS Pathog. 2013 Mar 14;9(3):e1003209. doi: 10.1371/journal.ppat.1003209 (PMC3597519; doi:10.1371/journal.ppat.1003209)
Supplement: Text S1 — Supporting text file containing the theoretical appendix, supporting experiments and statistics. (PDF) [file ppat.1003209.s009.pdf]

Supporting information for
"Evolution of virulence in emerging epidemics"

T.W. Berngruber, R. Froissart, M. Choisy and S. Gandon

S1. Theoretical evolutionary epidemiology

S1.1 Epidemiology

S1.2 Evolution

S1.3 Simulations

S2. Supplementary experiments and statistics

S2.1 Methods

S2.1.1 Life-history of fluorescently marked viral strains
S2.1.2 Quantifying competition in the free virus stage by marker specific qPCR

S2.2 First chemostat experiment

S2.2.1 Effects of initial prevalence and marker color on competition
S2.2.2 Test for the occurrence of mutations that compensate virulence

S2.3 Second chemostat experiment

S2.3.1 Competition at initial prevalence 1%, 10% and 99%
S2.3.2 Invasion of resistant host cells

S3. Supplementary references

S1. Theoretical evolutionary epidemiology

S1.1 Epidemiology

We derive below a model to describe the epidemiology and the evolution of the temperate bacteriophage λ in a population of fully susceptible *E. coli* bacteria (see **Figure 1,** in the main text for a schematic description of the life cycle). This model can be used to understand the dynamics taking place in a chemostat. In the absence of infection, the bacteria is assumed to reproduce at a rate r, and to die at a rate m (where m refers to the outflow rate of the chemostat). Density dependence is assumed to decrease the fecundity and to limit the bacterial density to the carrying capacity K. We assume that multiple virus strains may circulate. For each strain i we model both the dynamics of the density I_i of infected bacteria (provirus stage) and the density V_i of viral particles circulating in the medium (free virus stage). We assume that lysis of an infected bacteria releases a constant number B (burst size) of virus particles. Free virus may die at a rate m (where, again, m refers to the outflow rate of the chemostat) or adsorb to both infected and uninfected bacteria at a rate a. The adsorbed virus may enter the cell with a probability b, and, with the probability ϕ_i, it may integrate in the bacterial genome of the bacteria. Infected bacteria are reproducing at a rate ρ_i and the virus is vertically transmitted with probability δ (the fidelity of vertical transmission). For the sake of generality, we assume that with probability ϕ_{ij} bacteria infected with strain i can be superinfected with a strain j which replaces strain i. Infected bacteria may lyse when the virus fails to integrate into the bacterial genome, with probability $1-\phi_i$, but also after genome integration at a constant rate α_i (the lysis rate). The virulence of the virus (the mortality of the host induced by the virus) hence depends on both $1-\phi_i$ and α_i. The above described life cycle yields the following system of ordinary differential equations:

$$\begin{aligned}\dot{S} &= (rS+\rho_\circ(1-\delta)I)(1-N/K)-abSV-mS\\ \dot{I}_i &= \rho_i I_i\delta(1-N/K)+ab\phi_i V_i S-(\alpha_i+m)I_i+abV_i\textstyle\sum_j I_j\phi_{ji}-abI_i\sum_j V_j\phi_{ij}\\ \dot{V}_i &= ab(1-\phi_i)V_i SB+\alpha_i BI_i-mV_i-aNV_i\end{aligned} \qquad \text{(A1)}$$

The total density of infected bacteria is $I=\sum_i I_i$, and the total density of free virus is $V=\sum_i V_i$, and $N=S+I$. The frequencies of strain i are $p_i=I_i/I$ and $q_i=V_i/V$ in the provirus and in the free-virus stage, respectively. We use the following notations to refer to the value of the phenotypic trait z of the virus averaged over the provirus stage, $z_\circ=\sum_i p_i z_i$, or over the free-virus stage, $z_\bullet=\sum_i q_i z_i$.

The epidemiological dynamics of the total density of the virus (either in the provirus stage or in the free-virus stage) is thus:

$$\begin{aligned}\dot{S} &= (rS+\rho_\circ(1-\delta)I)(1-N/K)-abSV-mS\\ \dot{I} &= \rho_\circ\delta I(1-N/K)+ab\phi_\bullet SV-(\alpha_\circ+m)I\\ \dot{V} &= ab(1-\phi_\bullet)VSB+\alpha_\circ BI-mV-aNV\end{aligned} \qquad \text{(A2)}$$

The condition for a resident virus (with phenotypic traits α and ϕ) to generate an epidemic can be derived from the calculation of the basic reproductive ratio using the next-generation-matrix method [S1]. The parasite life-cycle can be decomposed into fecundity (matrix F) and mortality (matrix V) components:

$$F=\begin{pmatrix}\rho\delta\lambda & ab\hat{S}\phi\\ \alpha B & ab(1-\phi)\hat{S}B\end{pmatrix}$$
$$V=\begin{pmatrix}\alpha+m & 0\\ 0 & m+a\hat{S}\end{pmatrix}$$

where $\lambda = (1 - N/K)$ and $\hat{S} = K(r-m)/r$ refers to the density of susceptible bacteria before the introduction of the virus in the chemostat. The matrix F gives the rates at which new individuals appear in the provirus or in the free virus stages. The matrix V gives the rate at which these individuals die. The basic reproduction ratio is the spectral radius of the matrix $F\,V^{-1}$ which is:

$$R_0 = \frac{A+\sqrt{A^2-4abBS(m+aS)(m+\alpha)(\delta\lambda\rho(1-\phi)-\alpha\phi)}}{2(m+aS)(m+\alpha)} \tag{A3}$$

with $A = m\delta\lambda\rho + aS(\delta\lambda\rho + bB(m+\alpha)(1-\phi))$. The above expression can be readily used to find the parameter values allowing the virus to generate an epidemic in the chemostat (i.e. when $R_0 > 1$).

S1.2 Evolution

To better understand the evolution of the virus we focus next on the dynamics of the frequency of strain i in both the provirus (p_i) and the free-virus (q_i) compartments.

Using $\dot{p}_i = \frac{\dot{I}_i}{I} - p_i\frac{\dot{I}}{I}$, (A1) and (A2) we obtain:

$$\dot{p}_i = p_i\left(\overbrace{(\rho_i - \rho_\circ)\delta(1-N/K)}^{growth} - \overbrace{(\alpha_i - \alpha_\circ)}^{lysis} + \overbrace{abS\frac{V}{I}(\phi_i - \phi_\bullet)}^{genome\ integration} + \overbrace{abV(\phi_{\circ i} - \phi_{i\bullet})}^{superinfections}\right) + \underbrace{abV(q_i - p_i)\left(\frac{S}{I}\phi_i + \phi_{\circ i}\right)}_{\text{gene flow } between\ free\ virus\ and\ pro-virus\ compartment}$$

Similarly, using $\dot{q}_i = \frac{\dot{V}_i}{V} - q_i\frac{\dot{V}}{V}$, (A1) and (A2)we obtain:

$$\dot{q}_i = \overbrace{abSBq_i(\phi_\bullet - \phi_i)}^{failed\ genome\ integration} + B\frac{I}{V}\left(\overbrace{q_i(\alpha_i - \alpha_\circ)}^{lysis} + \underbrace{\alpha_i(p_i - q_i)}_{\text{gene flow } between\ pro-virus\ compartment\ and\ free\ virus}\right)$$

In the main text we consider a simpler scenario where we assume that only two strains are in competition (the avirulent wildtype and the virulent mutant), that infection does not affect the growth rate of the bacteria (i.e. $\rho_i = r$) and that superinfection is not possible (i.e. $\phi_{ij} = 0$). This yields equations (1) and (2). Note that in this model the only difference between the two virus strains occurs in the rate of genome integration and in the rate of lysis, and this is consistent with the properties of the mutant we are using (see **Figure S3** and [22]).

S1.3 Simulations

To generate specific predictions on the epidemiology and evolution of our system we simulated our model using parameter values given in **Table S1** below. Those parameters were chosen to match measures obtained in previous studies as well as our own measurements (see **Figure S3**). We explored the robustness of our theoretical predictions by allowing some variation on all the parameters affected by the mutation (i.e. the virulence phenotype): ϕ_R, ϕ_M, α_R, α_M. To do so we performed 10000 simulation runs, and for each run the values of these four parameters were drawn independently from a normal distribution with a mean and variance given in **Table S1**. In **Figures 2, S1 and S2** we plot all representations of these simulation runs and their median.

We further explored the potential effects of the evolution of bacterial resistance and virulence compensation in the virus using a modified version of the above model. In this new model we assumed that upon reproduction, susceptible bacteria could mutate with probability μ to a new type of bacteria, R, fully resistant to infection by the virus. Because resistance to λ requires the loss of a receptor, we further assumed that the resistance could induce a cost c on fecundity. In addition, we considered that the virus could mutate back and forth between the virulent and the avirulent phenotype. Our experimental method tracks the change in frequency of the fluorescent marker, and not the phenotype. These mutations would thus break the linkage between the marker and the virulence phenotype. To explore the effect of these mutations we allowed the virulence phenotype to change from i to j with probability ε_{ij}, but the tag always remains the same. This yields the following system of equations:

$$\dot{S} = (r(1-\mu)S + \rho_\circ(1-\delta)I)(1-N/K) - abSV - mS$$
$$\dot{R} = (r\mu S + \rho(1-c)R)(1-N/K) - mR$$
$$\dot{I}_{ii} = \big((1-\varepsilon_{ii})\rho_i I_{ii} + \varepsilon_{ji}\rho_j I_{ji}\big)\delta(1-N/K) + ab\phi_i V_{ii} S - (\alpha_i + m) I_{ii}$$
$$\dot{I}_{ji} = \big((1-\varepsilon_{ji})\rho_j I_{ji} + \varepsilon_{ij}\rho_i I_{ii}\big)\delta(1-N/K) + ab\phi_j V_{ji} S - \big(\alpha_j + m\big) I_{ji}$$
$$\dot{V}_{ii} = ab(1-\phi_i)V_{ii}SB + \alpha_i B I_{ii} - mV_{ii} - a(S+I)V_{ii}$$
$$\dot{V}_{ji} = ab\big(1-\phi_j\big)V_{ji}SB + \alpha_j B I_{ji} - mV_{ji} - a(S+I)V_{ji}$$

where I_{ij} refer to the density of bacteria infected by the virus with phenotype i and fluorescent tag j. Similarily V_{ij} refers to the density of free virus with phenotype i and fluorescent tag j. In addition we assume $= \sum_{i,j} I_{ij}$, $N = S + I + R$, $V = \sum_{i,j} V_{ij}$, $z_\circ = \sum_{i,j} \frac{I_{ij}}{I} z_i$ and $z_\bullet = \sum_{i,j} \frac{V_{ij}}{V} z_i$. Because we only considered 2 phenotypes (wildtype and virulent mutant) and 2 tags (the 2 fluorescent markers), this yields a system of 9 ordinary differential equations in total.

The epidemiological dynamics of the total density of the virus (either in the pro-virus stage or in the free-virus stage) is thus:

$$\dot{S} = (r(1-\mu)S + \rho_\circ(1-\delta)I)(1-N/K) - abSV - mS$$
$$\dot{R} = (r\mu S + \rho(1-c)R)(1-N/K) - mR$$
$$\dot{I} = \rho_\circ \delta I(1-N/K) + ab\phi_\bullet SV - (\alpha_\circ + m)I$$
$$\dot{V} = ab(1-\phi_\bullet)VSB + \alpha_\circ BI - mV - a(S+I)V$$

As above, we simulated the model to generate specific predictions on epidemiology and evolution when virulence compensation was possible (**Figure S1**) and when host resistance was allowed (**Figure S2**). In both cases we show that, although these mutations can affect the medium to long-term dynamics (after 24h), the short-term predictions discussed in the main text still hold.

Table S1. Model parameters with their definitions, values and units. The virulence phenotypes (ϕ and α) were sampled in a normal distribution $\mathrm{N}(\bar{z}, \sigma_z)$ with mean $\bar{z}$ and standard deviation σ_z in **Figure 2** and **Figures S1, S2**.

Parameter	Definition	Value	Unit	Experimental estimate	References
r	growth rate of uninfected cells	1.4	h^{-1}	$0.21\text{-}1.26$	S2
ρ	growth rate of infected cells	1.4	h^{-1}	$\rho \sim \mathrm{r}$	S3
K	carrying capacity	10^9	$cell$	$1\text{-}2{\cdot}10^9$	S4
δ	fidelity of vertical transmission	1	-	~ 1	S5
B	burst size	200	$virus \cdot cell^{-1}$	$9.7\text{-}255$	S6
m	dilution rate	0.75	h^{-1}	0.8	our study
a	adsorption constant	10^{-8}	$h^{-1} \cdot cell^{-1}$	$7.9\text{-}59{\cdot}10^{-8}$	S7
b	probability of fusion after adsorption	10^{-2}	-		
ϕ_R	probability of genome integration of λ	$\mathrm{N}(0.3, 0.03)$	-	0.6	our study
ϕ_M	probability of genome integration of λcI857	$\mathrm{N}(9{\cdot}10^{-2}, 9{\cdot}10^{-3})$	-	0.09	our study
α_R	rate of reactivation of the λ provirus	$\mathrm{N}(5{\cdot}10^{-3}, 5{\cdot}10^{-4})$	h^{-1}	$10^{-4}\text{-}10^{-3}$	S8,S9, our study
α_M	rate of reactivation of the λcI857 provirus	$\mathrm{N}(5{\cdot}10^{-2}, 5{\cdot}10^{-3})$	h^{-1}	$>10^{-3}$	S9 our study
μ	probability of mutation rate towards resistance	10^{-2}	-	$2.5{\cdot}10^{-3}$	S10
c	cost of resistance	10^{-2}	-	$4{\cdot}10^{-3}\text{-}10^{-2}$	S11
$\varepsilon_{ij} = \varepsilon_{ji}$	probability of mutation that compensate virulence of λcI857	10^{-2}	-	0.0038	S10

S2. Supplementary experiments and statistics

S2.1 Methods

S2.1.1 Life-history of fluorescently marked viral strains

The life-history traits affecting virus production (*PFU*), host growth (*CFU*) and lysogenization rate (*Lysogenized*) are presented in **Figure S3**. By ANOVA, we statistically tested the contribution of the factors *Strain* (λ or λcI857), *Temperature* (35 and 38°C) and *Color* (CFP or YFP) to the life-history traits *PFU*, *CFU* and *Lysogenized* (Figure S3). ANOVA on *PFU* (**Table S2.1**), *CFU* (**Table S2.2**) and *Lysogenized* (**Table S2.3**) revealed that *Strain*, *Temperature* and *Color* significantly affect all three life-history traits (except that lysogenization was only assayed at 35°C and its temperature dependence could not be determined). Even though the effect of *Color* is significant, the magnitude of its effect is several orders of magnitude lower than the effect of *Temperature* and/or *Strain*, as is visible by the percentage of sum of squares explained by each covariate (see **Table S2.1, S2.2 and S2.3, but also Figure S3**). Nevertheless, we experimentally controlled for the potential effect of color by carrying out each competition in 2 marker/virulence combinations (λCFP vs λcI857YFP and λYFP vs λcI857CFP).

Table S2.1. ANOVA for virus production (PFU/mL, ":" refers to interaction)

	Df	Sum Sq	Mean Sq	F value	Pr(>F)	% of total Sum Sq
Strain	1	4.1770e+22	4.1770e+22	348.7900	< 2.2e-16	31.9
Temperature	1	3.5768e+22	3.5768e+22	298.6682	< 2.2e-16	27.3
Color	1	8.4085e+20	8.4085e+20	7.0213	0.009140	0.6
Color:Strain	1	8.4107e+20	8.4107e+20	7.0231	0.009131	0.6
Color:Temp	1	9.7030e+20	9.7030e+20	8.1022	0.005202	0.7
Strain:Temp	1	3.5662e+22	3.5662e+22	297.7838	< 2.2e-16	27.2
Color:Strain:Temp	1	9.6471e+20	9.6471e+20	8.0555	0.005329	0.7
Residuals	120	1.4371e+22	1.1976e+20			11.0

Table S2.2. ANOVA for vertical transmission (CFU/mL, ":" refers to interaction)

	Df	Sum Sq	Mean Sq	F value	Pr(>F)	% of total Sum Sq
Strain	1	5.0789e+18	5.0789e+18	4041.1538	< 2e-16	76.2
Temperature	1	6.8333e+17	6.8333e+17	543.7086	< 2e-16	10.3
Color	1	6.7864e+15	6.7864e+15	5.3998	0.02379	0.1
Strain:Color	1	8.9076e+15	8.9076e+15	7.0876	0.01011	0.1
Strain:Temp	1	8.0037e+17	8.0037e+17	636.8400	< 2e-16	12.0
Color:Temp	1	8.5785e+15	8.5785e+15	6.8257	0.01152	0.1
Strain:Color:Temp	1	7.0762e+15	7.0762e+15	5.6304	0.02111	0.1
Residuals	56	7.0380e+16	1.2568e+15			1.1

Table S2.3. ANOVA for lysogenization (% lysogenized, ":" refers to interaction)

	Df	Sum Sq	Mean Sq	F value	Pr(>F)	% of total Sum Sq
Strain	1	21135.2	21135.2	2860.9730	< 2e-16	98.5
Color	1	44.7	44.7	6.0538	0.02031	0.2
Strain:Color	1	68.5	68.5	9.2691	0.00503	0.3
Residuals	28	206.8	7.4			1.0

S2.1.2 Quantifying competition in the free virus stage by marker specific qPCR

We quantified free virus particles by specific qPCR on the CFP and YFP genes. Details on the primers we used are given in **Table S3**. A test for the primer cross-specificity is presented in **Figure S4**.

Table S3. CFP and YFP specific primers (CFP and YFP specific nucleotides are in boldface).

Primer name	Specificity (Plasmid of origin)	Sequence
FCFP275	CFP (pDH3)	5'-ACAAAAGAATGGAATCAAAG**C**T**C**A**T**-3'
RCFP390	CFP (pDH3)	5'-CGAAAGGGCAGATTG**TGT**-3'
FYFP275	YFP (pDH5)	5'-ACAAAAGAATGGAATCAAAG**T**T**A**A**C**-3'
RYFP390	YFP (pDH5)	5'-CGAAAGGGCAGATTG**ATA**-3'

S2.2 First chemostat experiment

S2.2.1 Effects of initial prevalence and marker color on competition

To statistically test the effect of the *Initial Prevalence* treatment and *Color* on the competition dynamics, we performed an ANOVA on the data in **Figure 3B,C**. In order to account for repeated measurements we treated time as a random effect. Results show that the *Initial Prevalence* treatment significantly affects competitive dynamics in the provirus (**Table S4.1**) and in the free virus stage (**Table S4.2**). The effect of *Color* is, however, marginally significant (p=0.07) only in the provirus stage. More important, the magnitude of the *Color* effect is 50 times lower than that of the *Initial Prevalence* treatment, as is visible by the percentage of sum of squares explained by each covariate (see **Table S4.1 and S4.2**). Based on this result, we pooled the data from 2 marker/virulence combinations (λCFP vs λcI857YFP and λYFP vs λcI857CFP) in the first experiment (see **Figure 3**) and in the second experiment (see **Figure S6 and Figure 4)**.

Table S4.1. ANOVA table for the effect of initial prevalence and color on competition in the provirus stage with time treated as random effect (Data of Figure 2B, ":" refers to interaction)

	Df	Sum Sq	Mean Sq	F value	Pr(>F)	% of total Sum Sq
Initial Prevalence	1	17.0562	17.0562	186.9355	< 2e-16	43.55
Color	1	0.3013	0.3013	3.3027	0.07042	0.77
Initial Prevalence:Color	1	0.0004	0.0004	0.0045	0.94644	0.01
Residuals	239	21.8066	0.0912			55.67

Table S4.2. ANOVA table for the effect of initial prevalence and color on competition in the free virus stage (Data of Figure 2C, ":" refers to interaction)

	Df	Sum Sq	Mean Sq	F value	Pr(>F)	% of total Sum Sq
Initial Prevalence	1	58.817	58.817	251.6524	< 2.2e-16	34.7
Color	1	0.270	0.270	1.1569	0.282666	0.2
Initial Prevalence:Color	1	2.255	2.255	9.6486	0.002011	1.3
Residuals	463	108.214	0.234			63.8

S2.2.2 Test for the occurrence of mutations that compensate virulence

In the late phase of our experimental epidemics the virulent λcI857YFP and λcI857YFP could have accumulated mutations that compensate the cI857 mutation to reduce the cost of virulence. To test for the occurrence of such compensatory mutations, we calculated the number of free virus particles that are produced per infected cell (viruses/cell). Due to its virulence, the λcI857 mutant is expected to produce more viruses/cell than the λ wildtype and, hence, the ratio $(\text{virus/cell})_{\text{mutant}}$ divided by $(\text{virus/cell})_{\text{wildtype}}$ should be larger than 1. Indeed, this ratio is above 1 throughout most of the experiment (see **Figure S5**). We can therefore conclude that the virulent λcI857CFP and λcI857YFP remained significantly more virulent than λCFP and λYFP throughout most of the experiment even if compensatory mutations might have occurred.

S2.3 Second chemostat experiment

S2.3.1 Competition at initial prevalence 1%, 10% and 99%

To further explore the relation between the maximal benefit of virulence and initial prevalence we ran 6 additional chemostat competitions (1%, 10% and 99% initial prevalence each in 2 marker/virulence combinations). The observed competition dynamics in the pro-virus and free-virus stage suggest that the transient benefit of virulence decreases with increasing initial prevalence (see **Figure S6**). We further explore and test this possibility in **Figure 4** by extracting the maximal virulent/non-virulent ratios from the first 15h of **Figure S6** and plotting them directly against initial prevalence. By a linear model on the data of **Figure 4** we statistically tested for the effect of *Initial Prevalence* (1%, 10% and 99%) and *Viral Life-Stage* (provirus and free virus) on maximal virulence (**Table S5**). This analysis shows that maximal virulence significantly decreases between 1% to 10% and 10% to 99% initial prevalence both in the provirus and in the free virus stage. Furthermore, the maximal virulence is significantly higher in the free virus than in the provirus stage for all *Initial Prevalence* treatments and the interaction between *Viral Life-Stage* and *Initial Prevalence* is not significant ($F_{2,6} = 0.622$, $p = 0.56$).

Table S5: Linear Model analysis for the effect of initial prevalence and viral life-stage on the maximal benefit of virulence (Data from Figure 4). ('Intercept' corresponds to the free virus stage at *Initial Prevalence 1%.* Contrasts on *Initial Prevalence* are chosen so that *Initial Prevalence 1%* is compared to *Initial Prevalence 10% (3rd line) and Initial Prevalence 10%* is compared to *Initial Prevalence 99%* (4th line))

	Estimate	Std. Error	t value	Pr(>|t|)
Intercept	3.5573	0.2396	14.850	4.17e-07
Viral Life-stage	-1.7281	0.2396	-7.214	9.12e-05
Initial Prevalence 10%	-1.0231	0.2934	-3.487	0.008234
Initial Prevalence 99%	-1.7295	0.2934	-5.895	0.000364

S2.3.2 Invasion of resistant host cells

In the second chemostat experiment we observed a drop in the overall prevalence of fluorescent cells after 40h in chemostats 1,2,3,4 and 6, but not in chemostat 5 (see **FigureS7**). Initially we had 3 alternative explanations for this drop in prevalence: (1) Our chemostats were infected by a bacterium other than *E. coli*, (2) non-fluorescent cells carry a prophage which spontaneously deleted the fluorescent marker, (3) non-fluorescent cells carry no prophage, but have acquired resistance to infection by λ at the lambda receptor, lamB.

To rule out explanations (1) and (2) we cross-streaked colonies from each chemostat (t=60h) against the indicator strain λKH54h80ΔcI. Strain λKH54h80ΔcI infects *E.coli* cells

through the FhuA receptor and lyses cells which do not carry a prophage. All colonies from chemostats 1,2,3,4 and 6 were sensitive to the indicator strain (see **Figure S8**). This demonstrates that the invading cells are still *E. coli* and that the invading cells carry no prophage. Since the colonies from chemostat 5 carry a prophage, they are not lysed by the indicator strain. After eliminating explanations (1) and (2) it is therefore most likely that invading cells have acquired resistance at the lambda receptor, lamB. The fact that the invading resistant cells can still be lysed by the indicator strain which enters through FhuA receptor rather than the lamB receptor supports the view that the invading cells have acquired resistance in the original target of phage λ, the lamB receptor.

S3. Supplementary references

S1. Diekmann O, and Heesterbeek JAP. (2000) Mathematical Epidemiology of Infectious Diseases: Model Building, Analysis and Interpretation. Chichester: John Wiley and Sons.

S2. Andersen K, von Meyenburg K (1980) Are growth rates of Escherichia coli in batch cultures limited by respiration? J Bact 144(1):114-123. p.117.

S3. Lin L, Bitner R, and Edlin G. (1977) Increased reproductive fitness of Escherichia coli lambda lysogens. J. Virol. 21:554–559.

S4. Ausubel FM, Brent R, Kingston RE, Moore DD, Seidman JG et al. (1999) Short Protocols in Molecular Biology, 5th Edition: Vol.1 pp1-5. Current Protocols. Hoboken: Wiley

S5. Sly WS, Eisen HA, and Siminovitch L (1968) Host survival following infection with or induction of bacteriophage lambda mutants. Virology 34:112–127.

S6. Wang IN 2006. Lysis timing and bacteriophage fitness. Genetics 172:17–26.

S7. Shao Y, Wang IN (2008) Bacteriophage adsorption rate and optimal lysis time. Genetics 180:471–482.

S8. Little JW, Shepley DP, and Wert DW (1999) Robustness of a gene regulatory circuit. EMBO J 18:4299–4307.

S9. Zong C, So LH, Sepúlveda LA, Skinner SO, and Golding I. (2010). Lysogen stability is determined by the frequency of activity bursts from the fate-determining gene. Mol. Syst. Biol. 6:440.

S10. Drake JW, Charlesworth B, Charlesworth D, Crow JF (1998) Rates of spontaneous mutation. Genetics. 148(4):1667-86. p.1670.

S11. Bohannan, BJM, Travisano M, and Lenski RE. (1999) Epistatic interactions can lower the cost of resistance to multiple consumers. Evolution 53:292.
